# Genome-Wide Transcriptional Response of Avocado to *Fusarium* sp. Infection

**DOI:** 10.3390/plants13202886

**Published:** 2024-10-15

**Authors:** Michel Pale, Claudia-Anahí Pérez-Torres, Catalina Arenas-Huertero, Emanuel Villafán, Diana Sánchez-Rangel, Enrique Ibarra-Laclette

**Affiliations:** 1 Red de Estudios Moleculares Avanzados (REMAV), Instituto de Ecología, A.C. (INECOL), Xalapa 91073, Veracruz, Mexico; michel.pale@posgrado.ecologia.edu.mx (M.P.); claudia.perez@inecol.mx (C.-A.P.-T.); emanuel.villafan@inecol.mx (E.V.); 2 Investigador por México-CONAHCYT en el Instituto de Ecología, A.C. (INECOL), Xalapa 91073, Veracruz, Mexico; 3 Facultad de Ciencias, Universidad Autónoma de San Luis Potosí, San Luis Potosí 78295, San Luis Potosí, Mexico; catalina.arenas@uaslp.mx

**Keywords:** *Persea americana*, defense response, *Fusarium* infection, miRNAs

## Abstract

The avocado crop is relevant for its economic importance and because of its unique evolutionary history. However, there is a lack of information regarding the molecular processes during the defense response against fungal pathogens. Therefore, using a genome-wide approach in this work, we investigated the transcriptional response of the Mexican horticultural race of avocado (*Persea americana* var. *drymifolia*), including miRNAs profile and their possible targets. For that, we established an avocado–*Fusarium* hydroponic pathosystem and studied the response for 21 days. To guarantee robustness in the analysis, first, we improved the avocado genome assembly available for this variety, resulting in 822.49 Mbp in length with 36,200 gene models. Then, using an RNA-seq approach, we identified 13,778 genes differentially expressed in response to the *Fusarium* infection. According to their expression profile across time, these genes can be clustered into six groups, each associated with specific biological processes. Regarding non-coding RNAs, 8 of the 57 mature miRNAs identified in the avocado genome are responsive to infection caused by *Fusarium*, and the analysis revealed a total of 569 target genes whose transcript could be post-transcriptionally regulated. This study represents the first research in avocados to comprehensively explore the role of miRNAs in orchestrating defense responses against *Fusarium* spp. Also, this work provides valuable data about the genes involved in the intricate response of the avocado during fungal infection.

## 1. Introduction

MicroRNAs (miRNAs) are canonical non-coding small RNAs (20–24 nt) that are already known to silence gene expression, mostly through cleavage of target transcripts and translational repression and, in a few cases, by directing DNA methylation [1]. They are key plant development, growth, and reproduction regulators that also participate in abiotic or biotic stress responses [2,3]. Biotic stress responses are a complex network of molecular mechanisms that allow plants to deal with adverse conditions [4]. These defense mechanisms include molecular pattern-triggered immunity (PTI) [5,6] and effector-triggered immunity (ETI) [7]. They are activated upon recognition of pathogen-associated molecular patterns (PAMPs) [8] and pathogen effectors [9], respectively. Upon activation of these immune responses, plants undergo distinct cellular changes, such as reprogramming of secondary metabolism, production and detoxification of reactive oxygen species (ROS), biosynthesis of phytohormones, cell wall remodeling, and expression of resistance genes [10,11,12]. miRNAs play a crucial role in regulating these responses by modulating the expression of target genes involved in stress signaling pathways [3,13,14,15].

miRNAs can regulate hormone signaling pathways by targeting key genes; for example, in *Arabidopsis thaliana* (L.), during *Pseudomonas syringe* van Hall infection, flagellin detection (a bacterial elicitor) induces the *miR393* expression, which in turn silences target genes such auxin receptors *TIR1*, *AFB2*, and *AFB*, thus preventing/avoiding the pathogen proliferation [16]. Other miRNAs can fine-tune ROS levels by silencing genes encoding antioxidant enzymes or regulator genes involved in ROS production. This is the case of *miR398b*, which in rice, and in response to fungal infection, promotes the production of H_2_O_2_ through superoxide dismutase activity [17]. miRNAs can also modulate gene expression in cell wall synthesis and remodeling. This is the case of *miR397*, which negatively regulates the resistance of *Malus hupehensis* (Pamp.) to *Botryosphaeria dothidea* (Moug. ex Fr.) by modulating laccase 7 (LAC7), which is involved in lignin biosynthesis [18].

In cotton, during the infection with *Verticillium dahliae* Klebahn, *miR164* can directly cleave the mRNA of NAC100 to repress its expression level [19]. Downstream *miR164-NAC100* module mediates the expression of defense-related genes that contain the CGTA-box in their promoter [20], *e.g.*, the pathogenesis-related proteins (PR3, a chitinase) and the plant defensin PDF1.2, which is systemically activated by fungal pathogens and responds to methyl jasmonate but not to salicylic acid [19,21]. miRNAs can also reprogram the secondary metabolism, such as *miR858*, which has been implicated in defense responses against fungal pathogens and targets some *MYB* transcription factors involved in the biosynthesis of phytoalexins and phenolic compounds [14]. Evidence suggests that there is interaction between miRNAs and long non-coding RNAs (lncRNAs), a class of long RNAs that do not code for proteins but can regulate gene expression and modulate cellular processes by directly interacting with RNAs, DNA, or proteins [22,23]. It is even possible that some lncRNA are long non-coding primary microRNAs (lnc-pri-miRNAs), *i.e.*, lncRNA genes that produce miRNAs, or which can also be translated into small peptides with specific physiological functions [24,25,26]. For example, in tomato plants, a lncRNA functions as a decoy for miRNAs, sequestering these small RNAs and preventing them from binding to their original target, thus avoiding their regulatory activity. This mechanism modulates the response to pathogens [27,28].

The present knowledge about miRNAs has been achieved mainly through *in silico* approaches and functional characterization in model plant species and/or those of commercial interest, such as *A*. *thaliana*, rice, sugarcane, cucumber, strawberry, apple, or cotton [14,15,29,30,31,32,33]. While significant progress has been made in elucidating the roles of miRNAs in herbaceous plants, the study of miRNAs in woody species or those belonging to the magnoliids clade, an anciently diverged group of angiosperms plants that branched off before the split between monocots and eudicots [34]. This knowledge gap represents a significant opportunity for future research to explore the regulatory roles of miRNAs in tree growth, development, and stress responses, which could have profound implications for forestry or agriculture.

The avocado (*Persea americana* Mill) is a paleopolyploid species belonging to the Laurales order and magnoliids clade [34]. It is one of Mexico’s most commercially valuable species [35,36]. The principal commercial groves are monocultures of the Hass cultivar grafted on the *drymifolia* variety (*Persea americana* var. *drymifolia*) [37,38,39]. Despite the benefits that this variety confers to the grafts (greater tolerance to biotic and abiotic stresses), the presence of fungal diseases in crops prevails, including fusariosis [40,41,42]. The *Fusarium* genus includes cosmopolitan filamentous fungi species capable of infecting at least 81 commercially important plant species, including avocado [42,43,44,45]. *Fusarium* species easily penetrate plant roots and spread to vascular tissues, where they occlude xylem vessels and produce an arsenal of toxins and hydrolytic enzymes that kill host cells, resulting in wilt leaves, necrosis, and eventually plant death [40,46,47]. To date, only a few previous reports have explored transcriptional responses against fungal pathogens (including *Fusarium* species) in avocados [45,48,49,50,51]; thus, there is a lack of information in this research field (more if only the *drymifolia* variety is considered).

In response, in this work, the transcriptional response of roots of *Persea americana* var. *drymifolia* during *Fusarium* sp. infection were explored. We implemented a novel hydroponic pathosystem using avocado seedlings of *drymifolia* variety and we monitored the root infection at four time-set points (1-, 7-, 14-, and 21 days post-inoculation; dpi) and independent libraries of mRNAs and miRNAs were generated. Using a genome-wide approach, we identified differentially expressed genes (DEGs) and miRNAs (DEmiRNAS). This work consolidates one of the first reports exploring the miRNA-mediated transcriptional responses to a pathogenic agent on avocado crop.

## 2. Results

### 2.1. Symptoms of Fusarium sp. Infection in Avocado Seedlings

**Figure 1:**
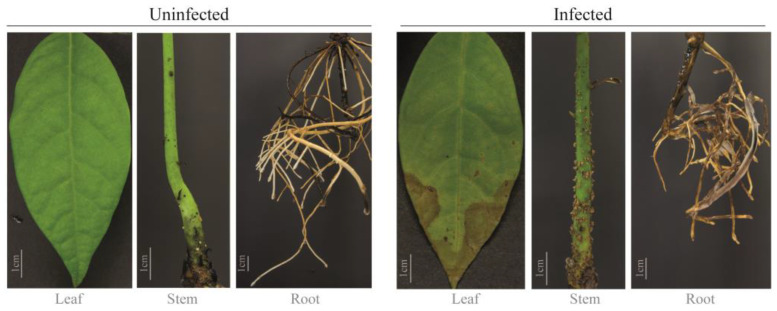
Symptoms of fusariosis in seedlings of avocado var. *drymifolia* at 30 dpi. Photography triptych on the left: leaf, stem, and root of uninfected plants (Control). Photography triptych on the right: leaf, stem, and root of infected seedlings. Infected plants were root inoculated with 1 × 10^6^ water-conidia suspension; control plants were treated with sterile water.

The fusariosis symptoms in avocado seedlings were progressive over time but were particularly evident at 30 dpi. In addition to wilting and chlorosis, necrotic areas were observed on leaves. Stems showed yellowing and polysaccharide accumulation, which is evidenced by the surface’s tylose cumulus formation and roots showed darkening and mycelium growth. In contrast, no symptoms were observed in control plants (Figure 1). To evaluate if the infection affects the physiology of the seedlings, we evaluated the growth of apical buds, stem height, or leaf loss; however, only in the growth of apical buds was a negative impact compared with control plants (Figure S1).

### 2.2. Improving Assembly and Annotation of the Avocado var. drymifolia Reference Genome

**Figure 2:**
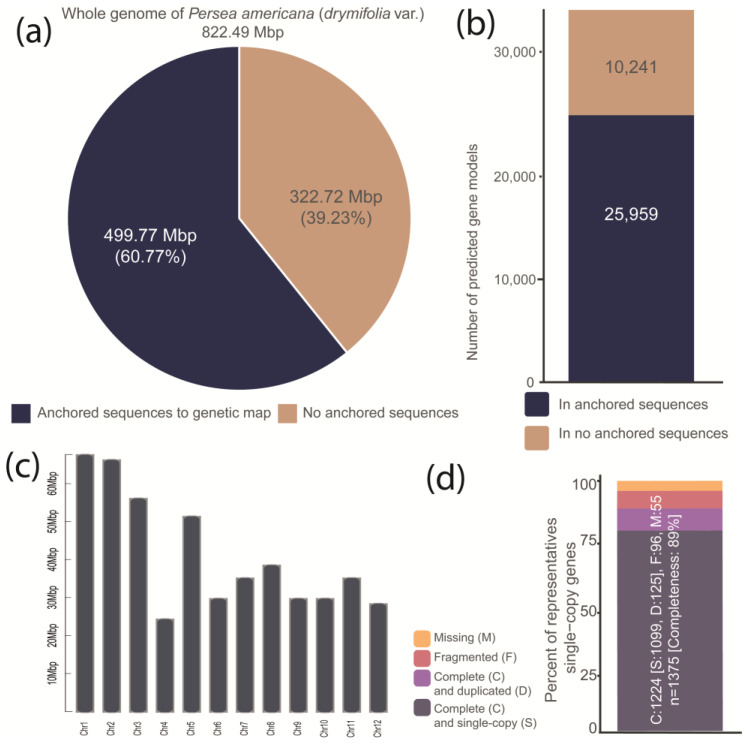
Schematic representation of some relevant genome metrics of avocado var. *drymifolia* genome. (**a**) Available assembled genome harbor 822.49 Mbp contained in a total of 7159 scaffolds. Input data from generating this new version were previously reported from Rendón-Anaya et al. [34] and were downloaded from GenBank. In total, 60.77% of the whole genome sequence (totaling 822.49 Mbp) was successfully anchored to the genetic map. A pie chart was used to visualize this information. (**b**) The gene set, which was predicted in both anchored and not anchored genomic sequences, comprises a total of 36,200 genes (25,959 and 10,241, respectively). (**c**) The anchored genome sequences to the genetic map are shown in a chromosome-scale graph. (**d**) Completeness estimated based on single copy orthologs shared between flowering plants from the dicotyledon clade (n = 1375). The bar’s colors represent the classes resulting from the BUSCO assessment.

We report an improved genome assembly from avocado var. *drymifolia* and its corresponding new annotation. This improved assembly includes sequence regions missing in earlier versions and the annotation of more than 10,000 new genes (36,200 in total; Table S1) resulting from combining methodologies used in gene models prediction/identification processes, *i.e.*, the *de novo* or *ab initio*, homology-based prediction, and/or endorsed by transcriptional evidence. The scaffolding process, based on the large-scale structure of the reference genome (the avocado Hass cultivar genome), allowed us to correct the order and the orientation of the more than 90,000 assembled contigs and reducing the 42,722 scaffolds from the earlier version to only 7159 (N50 of 30.33 Mbp; Table S2). In this new version, 98.8% of the contigs are part of some scaffold (Table S2), and 60.77% of the whole genome of avocado var. *drymifolia* is anchored into 12 pseudomolecules matching the avocado haploid chromosome number (Figure 2a–c). Based on its homology, with little more than 500,000 avocado transposable elements (TE) [34], 39.16% of the total sequence was recognized as repetitive DNA and softly masked as such (Table S3). With the presence of 1218 from the 1375 single-copy genes conserved in the embryophyte clade (https://busco.ezlab.org/frames/plants.htm, accessed on 23 October 2019), the completeness of the avocado genome was estimated at 89% (Figure 2d).

### 2.3. Avocado Responsive Genes to Fusarium sp. Infection

**Figure 3:**
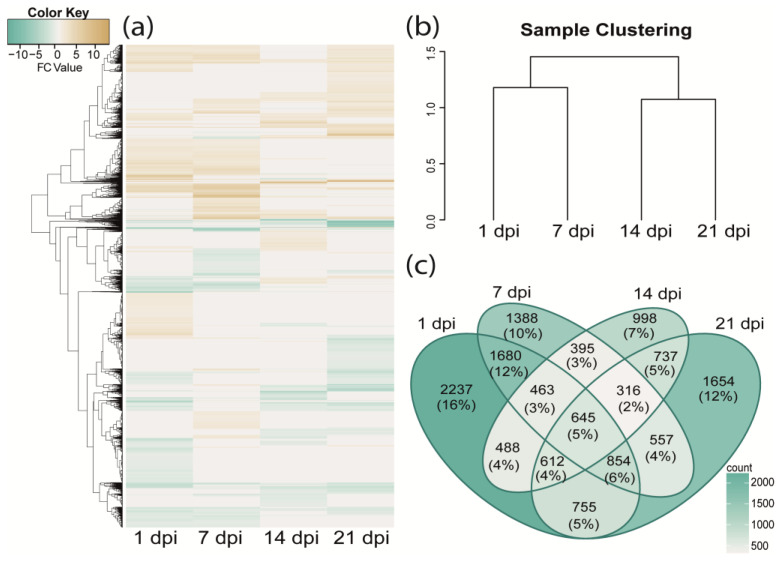
Genes of avocado var. *drymifolia* identified as differentially expressed (DE) in response to *Fusarium* sp. infection. (**a**) Heatmap of expression profiles showing differentially expressed genes (DEGs), (**b**) Hierarchical clustering tree that shows closeness (or similarity) between the distinct sampling points included in differential expression analysis, (**c**) Venn diagram which show DEGs identified on each sampling point. In parentheses, the percentage of the total represented by those DEGs shared or not, between each sampling point.

After the filtering and adapter trimming process, from the 16 sequenced mRNA libraries, a total of 419,170,882 high-quality paired-end (PE) reads (78.84% of the total) were obtained (around 26.2 million reads per library on average; Table S4). These libraries include two replicates from infected and uninfected (control) plants at 1-, 7-, 14-, or 21 dpi (see methods for details). As a result of the mapping process, close to 60% of these reads were aligned to the extended gene models predicted in the avocado var. *drymifolia* reference genome, 13,778 genes were identified as differentially expressed in at least one of the analyzed sampling points, and, in response to *Fusarium* sp. infection (changes which represent, in a significant way (*p*-adjusted value of ≤0.05), at least two-fold or greater (Log_2_FC = ±1); Table S5). A heatmap of DEGs showed gene expression levels across different sampling points (Figure 3a) while the hierarchical clustering analysis showed that DEGs at 1 and 7 dpi (early infections stages) were similar between them as well as 14 dpi and 21 dpi (late infections stages); no outliers were identified (Figure 3b). The time with the highest number of DEGs was 1 dpi with 2237, followed by 21 dpi and 7 dpi with 1654 and 1388 DEGs, respectively. The time set with the lowest number of DEGs corresponds to 14 dpi, with only 996 DEG (Figure 3c).

**Figure 4:**
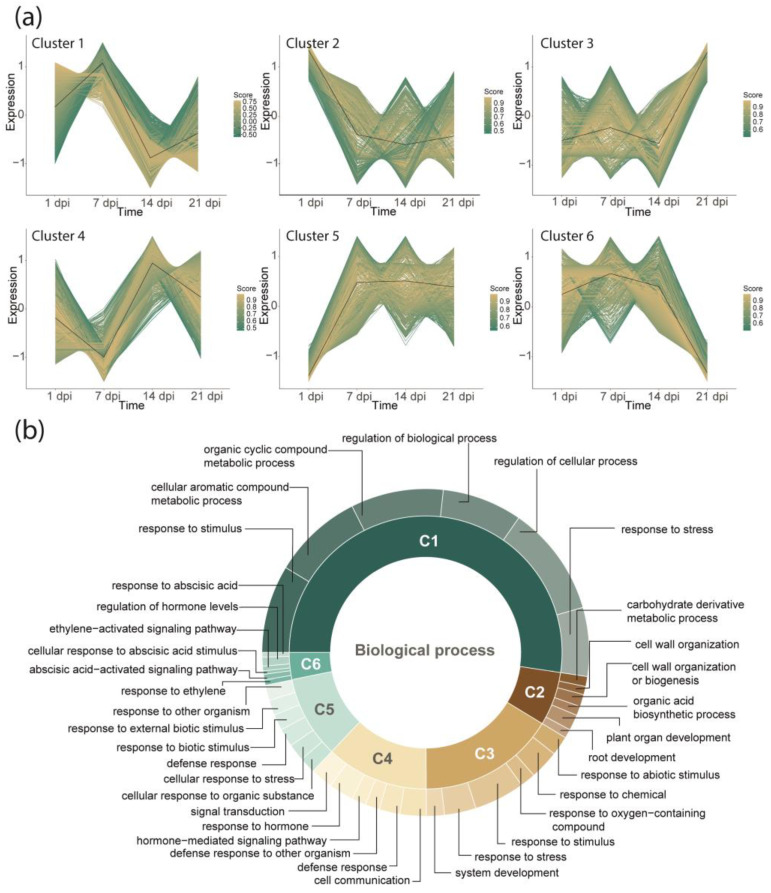
Clusters of DEGs formed based on their expression profiles and GO enrichment analysis, which shows the most representative functional categories for each cluster (six in total; C1–C6, respectively). (**a**) Clusters of DEGs with similar expression patterns responsive to *Fusarium* sp. infection. (**b**) Representative biological processes for each cluster generated).

Assisted by the *k*-means clustering analysis (see methods), the DEGs were grouped into six distinct clusters based on similarities of their expression patterns (Figure 4a, Table S5). Clusters one (C1) and two (C2) contain genes that reach their highest expression level at 1 or 7 dpi, respectively, and remain downregulated at the later times (14 and 21 dpi). Cluster three (C3) contains genes that are downregulated up to 14 dpi but increase their expression at 21 dpi. Similar behavior was observed in cluster four (C4), which contains genes that reach their highest expression levels at 14 and 21 dpi. Finally, cluster five (C5) includes genes that increase their expression levels from 7 dpi until the last sampling point. This contradicts the observation in cluster six (C6), where genes showed the highest expression levels from 1 dpi until 14 dpi and were downregulated at the last sampling point (21 dpi).

**Figure 5:**
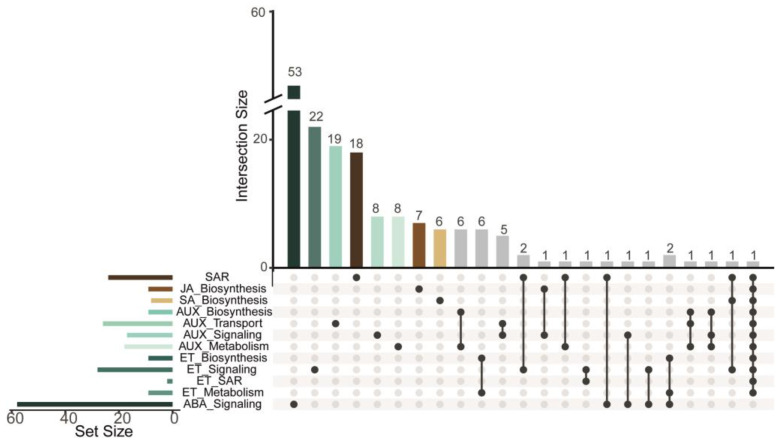
Main enriched hormonal processes in response to *Fusarium* sp. The figure shows the main phytohormones involved in the pathogenesis process and the number of genes involved in the regulated associated processes, which are biosynthesis, metabolism, transport, signaling, and regulation of SAR responses. Gray bars show those genes shared in multiple biological processes.

Table S6 lists all biological processes (GO-terms) enriched by the DEGs. Figure 4b shows the representative biological processes enriched by the genes belonging to each cluster formed based on their expression profile (C1–C6). Both C1 and C5 clusters contain genes that mainly enrich biological processes related to defense responses; some functional categories are shared and seem to be redundant or hierarchically related (*e.g.*, stress response (GO:0006950) and cellular response to stress (GO:0033554)). Despite this, others are specific, *i.e.*, are present in one or another of the clusters (C1 or C5, respectively). In C1, the top highly enriched categories are related to the biosynthesis of phenylpropanoids (*e.g.*, organic cyclic compound metabolic process (GO:1901360) and cellular aromatic compound metabolic process (GO:0006725)). In C5, the representative enriched categories are related to the regulation of the immune response, *e.g.*, response to biotic stimulus (GO:0009607), response to other organisms (GO:0051707), response to external biotic stimulus (GO:0043207), and cellular response to organic substance (GO:0071310). Interestingly, in C2, we identified genes related to root development: plant organ development (GO:0099402), cell wall organization or biogenesis (GO:0071554), carbohydrate derivative metabolic process (GO:1901135), organic acid biosynthetic process (GO:0016053), root development (GO:0048364), and cell wall organization (GO:0071555). In C3, DEGs enrich categories such as response and detoxification of ROS response to stress (GO:0006950), response to chemicals (GO:0042221), and response to oxygen-containing compounds (GO:1901700). Finally, C4 and C6 group genes are related to biosynthesis, response, and regulation of phytohormones. The major GO terms identified are as follows: cell communication (GO:0007154), response to hormone (GO:0009725), signal transduction (GO:0007165), defense response (GO:0006952), hormone-mediated signaling pathway (GO:0009755), and defense response to other organism (GO:0098542) in the C4 cluster; and response to abscisic acid (GO:0009737), cellular response to abscisic acid stimulus (GO:0071215), abscisic acid-activated signaling pathway (GO:0009738), response to ethylene (GO:0009723), regulation of hormone levels (GO:0010817), and ethylene-activated signaling pathway (GO:0009873) are the functional categories enriched by genes from the C6 cluster. According to the enriched categories, different phytohormones are active at different levels. The category with the greatest representativeness was abscisic acid (ABA)-mediated signaling with 58 genes identified, followed by biosynthesis, transport, signaling, and auxin (AUX) metabolism (51 DEGs identified). The next represented hormone was ethylene (ET) (also represented at the level of biosynthesis, signaling, and metabolism by 35 DEGs); this was expected as SAR is determined by the fact that plant inoculation with pathogenic flora is frequently accompanied by ethylene release. Genes involved in the biosynthesis of jasmonic acid (JA) and salicylic acid (SA) were also identified in the C4 and C6 clusters (15 DEGs). Additionally, we identified 18 genes involved in modulating SAR responses (Figure 5; Table S7).

### 2.4. Responsive miRNAs to Fusarium sp. and Target Identification

Identifying miRNA in plants is crucial because it plays a significant role in many biological processes, and several computational tools can identify it through *in silico* prediction. miRDeep2 v0.1.3, a tool that enables the accurate and reliable identification of new miRNAs [52,53], was used here to carry out the miRNA prediction in the avocado var. *drymifolia* genome (see methods). This software predicts miRNAs across the genome sequence based on some key features of the miRNA precursors (pre-miRNAs). These features, among others, involve the transcriptional evidence and expression level obtained from RNA-seq data, the expected DICER processing, the 21–22 nt long miRNA:miRNA* duplex stability, and the formation of a typical stable secondary structure with a minimum free energy estimated for the folded pre-miRNA hairpin. Thus, based on this strategy, a total of 57 miRNAs that fulfill the above-mentioned criteria were identified on the avocado var. *drymifolia* genome. Twenty-eight miRNAs resulted in homologs to miRNAs previously identified in other plant species, while the remaining twenty-nine were predicted as potentially new miRNAs. The names of the mature miRNAs assigned are shown in Table S8 and secondary structures for the potentially new miRNAs are shown in Figure S2. Typically, a miRNA begins with uridine (U) at its 5′ end [54,55]. Among the potential new miRNAs, only 12 of them share this characteristic, and they are *chr1_RaGOO_23292*, *Ctg1804_RaGOO_10559*, *chr12_RaGOO_20514*, *chr2_RaGOO_27520*, *chr9_RaGOO_46412*, *chr7_RaGOO_40014*, *chr1_RaGOO_21100*, *chr12_RaGOO_19719*, *Ctg3387_RaGOO_13007*, *Ctg2609_RaGOO_12391*, *Ctg0811_RaGOO_5704*, and *chr3_RaGOO_32118* (for details see Table S8).

As a result of Fisher’s exact test (see methods), eight miRNAs were identified DEmiRNAs in response to *Fusarium* sp. infection (Table 1); three of them with homologs previously identified in other plant species: *miR157d*, *miR166b*, and *miR166g* [28,56,57,58]; and five cataloged, according with the miRDeep2 criteria, as potential new miRNAs in avocado var. *drymifolia* (*chr11_RaGOO_17754*, *chr3_RaGOO_29551*, *chr4_RaGOO_33952*, *Ctg0811_RaGOO_5704*, and *Ctg0854_RaGOO_5920*). The miRNA *Ctg0811_RaGOO_5704* starts at 5′ with an Uracil (U) while *chr4_RaGOO_33952*, *Ctg0854_RaGOO_5920*, and *chr3_RaGOO_29551* start with an Adenine (A) and *chr11_RaGOO_17754* starts with a Guanine (G). All predicted sequences in these potential new miRNAs adopt the stable hairpin in the pre-miRNA secondary structure (Figure S2).

*In silico* analysis of target prediction of the eight miRNAs allowed us to identify 569 genes as potential targets of the DEmiRNAs (Table S9) and only 385 found a homolog in *A*. *thaliana* genome. From new potential DEmiRNAs, *chr11_RaGOO_17754* could regulate genes involved in distinct metabolic pathways or different biological processes, *e.g.*, participate in the downregulation of some LRR proteins, such RPK2 and AT1G53440, or in the downregulation of some cytochrome P450 enzymes, such CYP78A9 and CYP72A13. Also, it may regulate some protein transporters, such as potassium transporters POT10 and POT7. Regarding the *chr3_RaGOO_29551* miRNA, transcription factors such as *SPT6* and *AT3G18380*, as well as some transporters such as *ABCB15* and *AT2G23790*, were identified as their potential target genes. *chr4_RaGOO_33952* could regulate targets such as AT1G16860 and AT2G37195, which are membrane proteins, as well as transporters such as ABCB19, and even enzymes that catalyze the transfer of some molecules such as *e.g.*, SUVR5, UGT73B5, and AT5G24840. *Ctg0811_RaGOO_5704* regulates one Mitogen-activated protein, MAPKKK21, and two receptors, CLAVATA2 and AT1G53440. Finally, *Ctg0854_RaGOO_5920* possibly regulates transporters such as ABCG36, ABCB18, NIPA3, AT4G32510, and FOLT1. Besides, this miRNA might also regulate the Auxin Response Factors ARF16 and ARF2.

**Figure 6:**
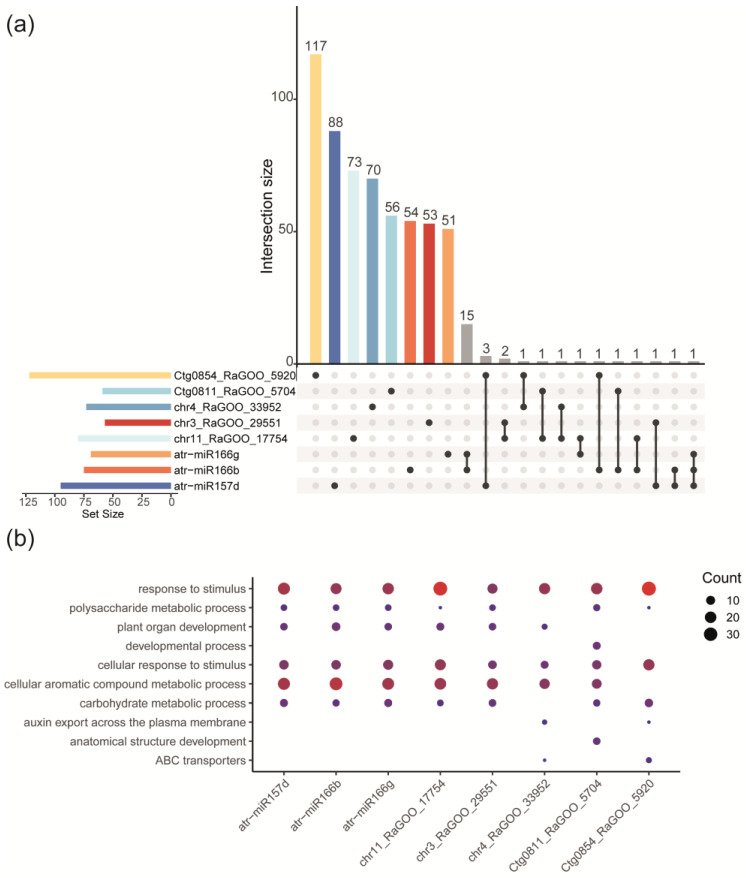
DEmiRNAs responsive to *Fusarium* sp. infection and the biological processes (BP) regulated by them. (**a**) UpSet plot of identified DEmiRNAs representing the number of target genes associated with each of them. (**b**) Bubble plot representing the main BP in which the associated targets of each identified DEmiRNA could intervene. The figure in the (**b**) panel was generated only considering the annotated target genes.

As shown in Figure 6a, *Ctg0854_RaGOO_5920* could regulate the largest number of target genes (76 annotated unique genes). On the other hand, *miR166b* and *Ctg0811_RaGOO_5704* are the DEmiRNAs with the smaller number of target genes (32 and 33 annotated unique genes, respectively; Figure 6a). Some DEmiRNAs seem to share target genes (Figure 6a; gray bars); this is the case of *miR166b* and *miR166g*, which, according to the performed analysis, share the greatest number of target genes identified (15 target genes in total: *AT1G05670*, *AT1G52150*, *AT1G53840*, *AT1G65790*, *AT2G34710*, *AT2G34710*, *AT2G34710*, *AT3G53920*, *AT4G37170*, *AT5G01530*, *AT3G04650*, *AT3G25640*, *AT5G43680*, *AT5G60690*, and *AT5G60690*), followed by the duo *Ctg0854_RaGOO_5920* and *miR157d*, which share three target genes (*AT3G44730*, *ATKP1*, and *AT5G42940*). *chr11_RaGOO_17754* and *chr3_RaGOO_29552* share two target genes, only one with annotation *AT4G03100*. Another pair of miRNAs that share target genes are the duo *miR166b*–*chr11_RaGOO_17754*, which share a single target, *MPSR1*. *Ctg0854_RaGOO_5920*–*chr4_RaGOO_33952* share *AT2G43120*; *miR157d*–*chr3_RaGOO_29551* could regulate *BHLH60*; *Ctg0854_RaGOO_5920*–*miR166b* share *SPA2*; *miR166g*–*chr11_RaGOO_17754* to *CLV2*; *miR157d*–*miR166b* target *AT4G22190*; *Ctg0811_RaGOO_5704*–*chr11_RaGOO_17754* share *BOLA2*; *chr11_RaGOO_17754*–*chr4_RaGOO_3395* to *DGS1*. In addition, *Ctg0811_RaGOO_5704*–*miR166b* could regulate AT5G50780. Finally, only one gene, *HDA05*, could be regulated by three DEmiRNAs (*miR157d*, *miR166b*, and *miR166g*).

**Figure 7:**
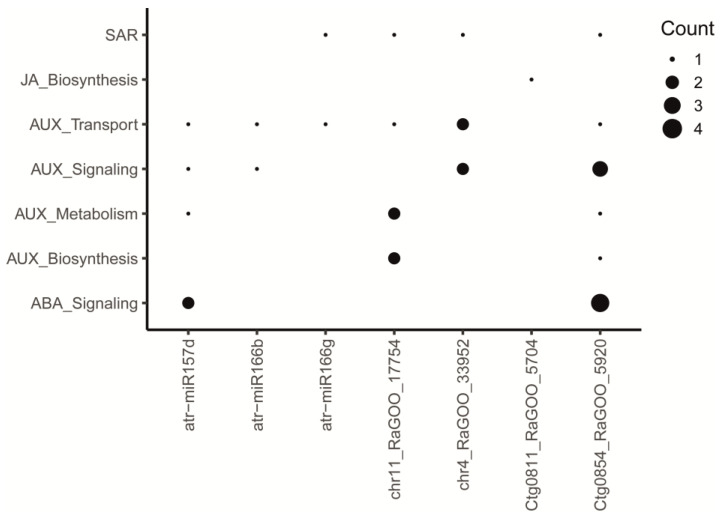
Involvement of DEmiRNAs in phytohormone regulation. The bubble plot illustrates the primary phytohormones and their respective roles, including biosynthesis, transport, metabolism, and involvement in SAR responses. It also indicates how the identified DEmiRNAs might intervene in these processes.

Functional enrichment analysis was performed by inheriting the GO annotation from the *A. thaliana* homologs to the predicted 385 miRNA target genes in the avocado var. *drymifolia* genome (Table S10). Interestingly, biological processes may be modulated by the target genes stand out response to stimulus (GO:0050896), cellular response to stimulus (GO:0051716), carbohydrate metabolic process (GO:0005975), auxin export across the plasma membrane (GO:0010315), and polysaccharide metabolic process (GO:0005976) (Figure 6b). Also, we found that DEmiRNAs modulate the biosynthesis of some phytohormones, their transport and signaling pathways, and SAR responses through action over their predicted targets (Figure 7; Table S11). The avocado DEmiRNAs could modulate the auxin biosynthesis by regulating the expression level of some genes, such as *AT5G20960*, *AT5G43890*, and *AT4G37750*. Target genes, such as *AT1G05680*, *AT5G20960*, *AT5G43890*, and *AT4G37750*, could modulate auxin metabolism, while *AT1G27340*, *AT1G78570*, *AT1G59870*, *AT1G78570*, *AT2G17800*, *AT3G62150*, and *AT5G47440* could modulate auxin signaling. Finally, the AUX transport could be regulated through target genes such as *AT1G68710*, *AT1G78570*, *AT3G02260*, *AT3G28860*, *AT1G59870*, *AT1G78570*, and *AT3G62150*. Concerning JA biosynthesis, it may be modulated by downregulating the *AT4G08850* gene. Finally, target genes such as *AT2G46370*, *AT1G19250*, *AT1G59870*, and *AT5G60900* seem to be involved in SAR responses.

## 3. Discussion

The defense response of avocado against fungi (and oomycetes) pathogens has been previously explored in different pathosystems using the whole plant or a specific tissue [45,48,49,59,60,61]. Only a few reports exist in relation to pathogens of the *Fusarium* genus [45,62,63,64,65]. Except for *Fusarium* species associated with the ambrosia beetles, it is known that most *Fusarium* infections begin with root colonization and spread through the xylem [66,67,68,69,70]. Here, we developed a practical and reproducible hydroponic pathosystem that maintains constant reflux of fungal inoculum in root tissue. This pathosystem allowed us to control different environmental variables that could intervene in our data and avoid extra damage caused by root manipulation. The symptoms developed by infected seedlings correspond to a typical fusariosis disease (Figure 1), which coincides with previous reports [71]. Interestingly, at later times post infection, the stem showed the presence of tylose cumulus, which was abundant in the near zone to the root (Figure 1). This symptom of the disease has been previously reported as a response of avocados against infection of distinct fungal pathogens, including some species of the *Fusarium* genus [45,72,73]. The tylose deposition, which tends to swell, blocks the xylem, and is a defensive mechanism used to restrict the spread of pathogens, especially fungi [72]. Finally, the root tissue of infected avocado plants exhibits darkened areas and evidenced mycelium growth like in a previous report in citrus plants [43] (Figure 1).

Despite advances in sequencing technologies currently allowing the generation of data collections at vastly decreased costs, it has been proven that improving a highly fragmented genome assembly is often possible by iterative mapping of short reads and without the need to generate new data [74]. The methodology implemented in this study, which consists in first ordering and orienting the contigs based on a reference genome and filling the gaps using iterative mapping of short reads (see methods and results above), was successful and allowed us to generate an improved and less fragmented version of the avocado var. *drymifolia* genome used in the previous version by Rendón-Anaya in 2019 [34]. The integrity of the new version of the avocado var. *drymifolia* genome (822.49 Mbp in length, 7159 scaffolds, N50 = 650 Mb, BUSCO completeness = 86.6%), now anchored to the chromosomes using a genetic map, is comparable to the two genomes available from the Hass cultivar [34,75].

In this work, we analyzed the root system because, as we already discussed, it is the first tissue that has contact with the fungus and forms a central axis in mediating various essential processes for the plant [76,77,78]. The 13,778 protein-coding genes that respond to fungus infection and which were identified as differentially expressed were grouped based on their expression profile (Figure 3a). Previous studies that analyzed transcriptional responses of avocado to some fungus pathogens are mainly limited to early stages of the disease [49,50,51,59,79]; in contrast, our analyses consider both early and late stages of disease (*i.e.*, 1 and 7 and 14 and 21 dpi, respectively). The higher number of DEGs in the early stages compared to the later ones may be attributed to the events in the early stages of infection, which may be mediated by pathogen recognition and immediate responses to overcome it and prevent its establishment [80,81,82]. This has been evidenced in previous studies, for example, in bananas, where there is a higher number of DEGs within the first 5 h of infection, decreasing over time (up to 25 hpi) [83]. Similarly, in a study conducted on avocado stems, the initial time analyzed (1 dpi) resulted in the highest number of DEGs in response to the pathogen *Fusarium kuroshium* (F. Na, J.D. Carrillo & A. Eskalen ex Sand.-Denis & Crous) O’Donnell, Geiser, Kasson & T. Aoki in contrast with later times [45].

We observed that roots from infected plants were smaller in size than controls; they also presented darkened areas and the establishment/growth of mycelium on its surface. Related to the smaller size, roots of *A*. *thaliana* infected with *Fusrium oxysporum* Schltdl exhibited significant suppression of genes related to plant growth, including genes associated with the cell cycle, cell-wall organization, plant-type cell-wall biosynthesis, and microtubule-based processes [84]. In this work, a set of genes of cluster 2 (C2) enriched the “root development” functional category (Figure 4b). We also found genes involved in auxin biosynthesis and transport, and some additional responses mediated by this phytohormone. This is consistent because AUX (mainly the Indole-3-Acetic Acid; IAA), a key plant hormone, is involved in root development processes, acting as an endogenous regulator of the root by modulating primary root growth and lateral root formation [85,86]. TAR2 and YUCCA (a TRYPTOPHAN AMINOTRANSFERASE RELATED 2 and a flavin monooxygenase protein, respectively) are important for IAA formation from tryptophan [78,87]. Additionally, we found genes involved in polar AUX transport, *e.g.*, ATP-dependent pumps (ATP-binding cassette (ABC) transporters), auxin influx transporters (AUX/LAX genes), and PIN auxin efflux carriers [88,89]. The involvement of AUX in the response to pathogens has been characterized in models such as rice and even avocado, where its participation was first suggested based on GO enrichment analysis [51,90].

Modifications in root structure could also be affected by flavonoid metabolism. This pathway is linked to the tissue lignification process because the lignin biosynthesis starts with the phenylpropanoid pathway, which can also generate flavonoid precursors [91,92]. Lignin is a highly recalcitrant phenolic polymer and can be deposited in the plant cell’s secondary wall, acting as a defense mechanism against pathogens [92,93]. This is consistent with the finding of some DEG-codifying proteins such as TT10, 4CL, or some MYB transcription factors. TT10 is an enzyme involved in flavonoid biosynthesis, and it has been proven to contribute to root elongation in *A*. *thaliana* [94]. The gene *4CL* (encodes a 4-Coumarate: CoA Ligase) catalyzes flavonoid and lignin precursor synthesis [91,95]. Finally, MYB4 acts as a regulator of MYB7, and both encode for MYB domain proteins involved in flavonoid biosynthesis and are predominantly expressed in roots [96]. For example, in *A*. *thaliana*, *RPP* genes (mainly *RPP8*) are induced in response to *F*. *oxysporum* infection in the early stages (12–96 hpi) [84].

The functional categories enrichment analysis shows that Gene Ontology (GO)-terms related to “defense responses” are enriched mainly by DEGs grouped into the C1 and C5 clusters. According to the *k*-mean clustering analysis, genes in C1 exhibit high levels of transcripts mainly during the early stage of infection (*i.e.*, at 1 and 7 dpi), and in C5, the DEGs reach their highest transcript levels at 7 dpi, and they remain upregulated until the last sampling point (21 dpi). The enriched functional categories (response to stress, response to biotic stimulus, defense responses, and response to another organism) have been reported to be enriched by DEGs in distinct plant–pathogen interactions studies such as *Medicago sativa* L.–*Fusarium proliferatum* (Matsush.) Nirenberg ex Gerlach & Nirenberg, *Cucumis sativus* L.–*Corynespora cassiicola* (Berk. & M.A. Curtis) C.T. Wei, *Musa acuminata* Colla–*F. oxysporum* f. sp. *cubense*, *Persea americana* cv. Hass–*F*. *kuroshium*, *P*. *americana*–*Phytophthora cinnamomic* Rands, *Zea mays* L.–*Fusarium verticillioides* (Sacc.) Nirenberg, and *Malus domestica* (Suckow) Borkh. –*F*. *proliferatum*, among others [33,45,79,97,98,99].

Many genes grouped into C1 and C5 are involved in pathogen recognition involving resistant proteins (R proteins). To date, only a few R proteins have been identified, *e.g.*, RPP13 was identified as a responsive R protein in avocado during the early stages of the *P*. *cinnamomi* infection [51], and, in a more recent study, four NLR-type disease resistance proteins were differentially expressed in response to *F*. *kuroshium* infection [45]. In this study, we identified at least eleven R proteins responsive to *F*. *kuroshium* infection (AT1G50180, AT1G53350-RPP8L2, AT3G50950-RPP13L4, AT4G19050, AT4G26090-RPS2, AT4G27190, AT5G05190-EDR4, AT1G53350-RPP8L2, AT2G34930, AT3G46730-RPP13L3, AT4G33300-ADR1-L1), including the RPP13 protein. These results are consistent with other previous studies in which the important role that R proteins play in early responses to *Fusarium* sp. infection [100].

In the case of transcription factors (TFs), we found different members of the WRKY family (*e.g.*, WRKY4, 7, 9, 13, 14, 18, 40, 44, 55, and 69), which have been previously reported as regulators of immune responses associated with both PTI and ETI [101,102]. WRKY40 can regulate the hypersensitive response (HR) in pepper against *Ralstonia solanacearum* (Smith) Yabuuchi *et al*. and modulates the expression of PR proteins, where its overexpression acts as a negative regulator in infection [102,103]. Another example of TFs are those members of the NAC family (NAC021, 035, 043, 073, 078, and 082), which have also been shown to be involved in pathogenesis processes [103,104,105]. They act as positive or negative regulators of immunity in plants, as well as regulators of the HR or targets of effectors from some pathogens [105]. This type of TFs has been identified as responsive in plant transcriptomes during a pathogenesis event caused by a fungus [51,106]. Additionally, MYB TFs were also identified (MYB3, 12, 46, 88, 93, and 111). These types of TF participate in the response to abiotic and biotic stress [107,108]. They have also been reported as responsive to pathogen infection in apple, maize, and pepper [69,98,109,110].

C4 and C6 clusters were of particular interest, as they contain enriched categories related to phytohormone processes such as biosynthesis and signaling. The most enriched category was ABA-mediated signaling (Table S7). This phytohormone is a key point for regulating various physiological processes in plants [111]. It can act as either an enhancer or inhibitor of root development and architecture [112]. ABA can intervene in the regulatory pathway of AUX by downregulating the expression of ARF (Auxin Response Factors) genes and thus influence the development and defense response processes [113,114]. ARF2 (encodes an Auxin Response Factor protein) is an AUX-responsive gene that is up-regulated in response to ABA, promoting primary root growth in coordination with *PIN* genes and *YUCCA* genes [88,112,115]. Both *ARF2* and *YUCC4* (also called *YUCCA4*) were identified as responsive to *Fusarium* sp. Dysregulation of ABA signaling could impact the expression of *ARF* genes, such as *ARF2*, which in turn influences the activity of AUX-responsive genes and root growth.

Additionally, it can function as a regulator (antagonistic or synergistic) of other phytohormones in a process known as crosstalk [116,117]. For example, it is already known that the interconnection between ABA and ET is highly dynamic [118,119]. An example of this is ABA’s activity as an antagonist of EIN3 (Ethylene Insensitive 3 TF), allowing the repression of ABI4 (ABA Insensitive 4 TF), resulting in the activation of VTC2 (a GDP-l-Galactose Phosphorylase) and causing an accumulation of ROS [114,120]. Both *EIN3* and *VTC2* are differentially expressed in response to the pathogenesis process. ABA activity on this pathway may be associated with the transcriptomic data from C3, which is evidenced by a response to ROS accumulation. ABA can also intervene in crosstalk with JA through genes involved in ABA signaling, such as *PYL* (an ABA sensor), *ABI*, or *ABF* (ABA-responsive TF) and genes of the JA signaling machinery, such as TIF (a TF responsive to JA), JAZ (a jasmonate ZIM-domain protein), and MYC (a TF responsive to JA) [114,119,121]. In response to the pathogen, we found the *PYL4* and *MYC2* genes were involved in the crosstalk between ABA and JA, described during biotic and abiotic stress [118]. ABA involvement in the regulation of defense responses in plants has been evidenced in some plant species such as *A*. *thaliana*, *Pinus radiata* D.Don, *M*. *domestica*, *Z*. *mays*, and avocado in response to pathogens such as *P*. *cinnamomi*, *Fusarium* spp., and bacteria like *P*. *syringae* [51,106,109,122,123,124].

In avocado root, we also identified genes related to SAR. This response involves producing and mobilizing defense signals, such as SA, throughout the plant [125,126,127,128]. The accumulation of SA and the activation of specific signaling pathways prepare the plant to respond more effectively to future infections [129]. This response is prolonged and can persist for several weeks or even months after the initial infection, providing long-lasting protection against a variety of pathogens [130]. Examples of key genes in this response include the *WRKY33* TF, which interacts with phytohormone signaling pathways, particularly the SA pathway, which is central to SAR. SA induces *WRKY33* and acts downstream of SA signaling to regulate the expression of SA-responsive genes [131,132]. This interaction contributes to establishing SAR and the priming of defense responses. WRKY33 also participates in crosstalk between different defense signaling pathways, such as those mediated by JA and ET [114,117,133]. We also identified genes such as *FLOWERING LOCUS D* (*FLD*). This gene has been characterized as necessary to promote resistance through SAR [134]. It is inferred to participate in the long-distance perception of SAR-mediated signaling, promoting the accumulation of SA and the activation of genes such as *PR1* (Pathogenesis-Related 1) and regulating *WRKY* TFs [135]. FLD is also capable of mediating SAR responses through a gene pathway involving FVE (a homolog of the mammalian retinoblastoma-associated protein), a gene responsive to *Fusarium* sp. and categorized within the genes involved in SAR responses, and whose expression levels increase during a SAR response [136]. Similarly, Elongator subunit 2 (ELP2), identified as responsive, intervenes in SAR regulation. Plants in which the *ELP2* gene is mutated compromise SA signaling, which is essential for SAR and increases susceptibility to pathogens [137]. SA is necessary for SAR to occur. SA can be synthesized through two pathways, one mediated by the activity of the enzyme isochorismate synthase (ICS) and the second by phenylalanine ammonia lyase (PAL) [93,137]. These genes were identified as responsive in the rapeseed transcriptome, along with one of the main SA transporters in plants, ENHANCED DISEASE SUSCEPTIBILITY5 (EDS5) [138].

In this work, we were also interested in analyzing miRNAs because they provide insight into how they modulate responses against *Fusarium* sp. We identified eight DEmiRNAs, five predicted as potentially new miRNAs and the three remaining, *miR157*, *miR166b*, and *miR166g*, were conserved in other plant species. Together, these DEmiRNAs could regulate 569 targets involved in distinct biological processes. During plant–pathogen interaction, miRNAs play an important role mediating the transcriptional responses to avoid pathogen proliferation. In general, most enriched functional categories are involved in metabolic process and plant development (Figure 6; Table S9). In comparison with other biological models, there exist some similarities with our results, *e.g.*, in maize, in response to *F*. *verticillioides* infection, cellular and metabolic processes are the most representative categories as well as in banana cultivars exposed to *F*. *oxysporum* disease [139,140]. The phenylpropanoid metabolic process is a relevant category in our data set of gene targets, which is also relevant in maize–*F*. *verticillioides* infection [140]. Interestingly, those DEmiRNAs conserved at an evolutionary level in avocados regulate different target genes to those reported in other plant species; this phenomenon, in which the same miRNAs do not regulate homologs target genes in different species, has been previously described [28,56,57,58,141,142].

For *miR157*, it may regulate the activity of the LYK3 (a LysM-containing receptor-like kinase). This gene is involved in responsive gene induction upon recognition of elicitor molecules such as flagellin or chitin [143]. It also acts as a negative regulator of infection; for example, in *A. thaliana*, loss of LYK3 function shows reduced area of lesions and increased relative expression of defense-related genes in a crosstalk response with ABA [144]. *miR166b* and *miR166g* are members of the same family and share most target genes (Figure 6a), like the *miR156* family, which shares targets with the *miR157* family due to their high sequence homology [56,145]. In our analysis, *miR166b* may intervene in the regulation of MYB4 TF involved in flavonoid biosynthesis [96] and represented as a DEG. Finally, *miR166g* potentially regulates the expression of the *JAR1* gene, which is essential for generating the active form of JA, JA-Isoleucine (JA-Ile) [146]. This molecule is crucial for signaling responses mediated by this phytohormone [83,147,148,149].

The variability of miRNAs can be attributed to several factors, some related to the origin of MIR genes (*i.e.*, gene inverted duplication, *de novo* origin, or action of miniature inverted-repeat TE) [150]. Moreover, the nature of plant genomes, characterized by frequent gene duplications, rearrangements, loss events, and TE activity [151], could contributes to the divergence of miRNA sequences across plant species [152] and provides insights into gene regulatory networks as adaptive strategies employed by plants to avoid environmental challenges. Thus, since no homologs to the putative new miRNAs were found from other species in the miRBase database, we considered that they are lineage- or avocado-specific. Take into account that the genome from only a few species of the Magnoliidae clade has been sequenced (the largest clade of flowering plants outside monocots and eudicots, and which exhibit some features like “Early Angiosperms” [153]), and even fewer species are available (less than ten in GenBank) from the Laurales order. The secondary hairpin loop structures of the pre-miRNA sequences, along with the high negative minimal folding energy (MFE), were “reliable criteria” for the computational identification of these miRNAs [154]. Besides, especially in plant species, the feature of nearly perfect or perfect complementarity of miRNAs to their target mRNA sequences also allows the reliable computational prediction of miRNA target genes [155,156].

The putative avocado-specific miRNAs are involved in the regulation of relevant biological processes that need to be modulated as part of the biotic stress response. For example, the regulation of AUX biosynthesis and metabolism occurs by the miRNA *chr11_RaGOO_17754*, the target of which is the YUC5 gene. As mentioned above, *YUCCA* genes are important for AUX biosynthesis [78,87]. Notice that the AUX metabolism is affected by miRNA dynamics in other plants during a pathogenesis event against different pathogens, including *Fusarium* spp. and bacteria [124,157,158,159]. *chr11_RaGOO_17754* targets the gene FMO1 (a Flavin-dependent monooxygenase), which plays a crucial role in SAR and positively regulates this response, modulating SA biosynthesis and downstream defense coordinated responses [160,161]. *chr4_RaGOO_33952* could regulate *PEN3*, a member of the ATP-binding cassette (ABC) transporter family, which contributes to SAR defense by coordinating the transport of antimicrobial compounds. Plants with loss of *PEN3* function compromise the defense responses [162,163]. On the other hand, *Ctg0854_RaGOO_5920* could regulate the *RLK1* gene, which acts as a crucial mediator in SAR through the recognition of pathogen-derived signals and initiating intracellular signaling cascades coordinating the activation of defense genes [164]. Interestingly, the regulation of SAR responses by miRNA activity has been previously reported in other plant species [157,165].

**Figure 8:**
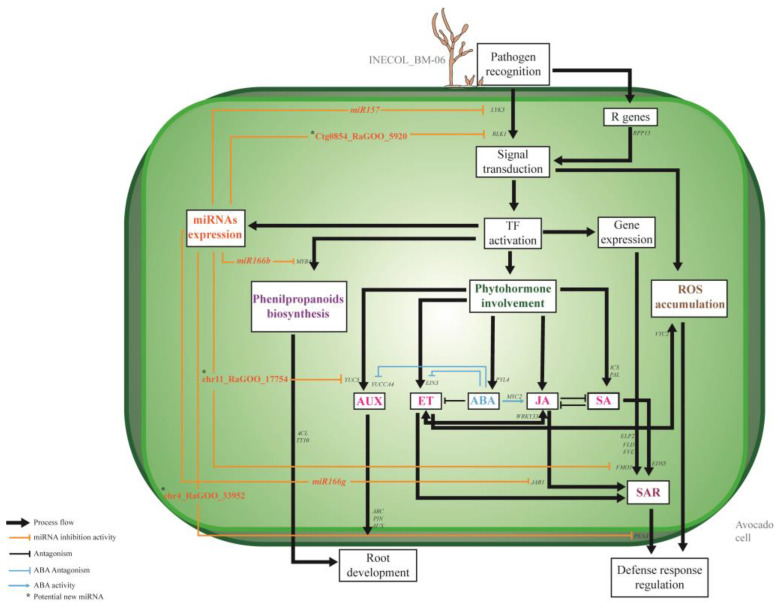
A schematic representation of the innate immune system model by which avocado var. *drymifolia* seeks to counteract the *Fusarium* sp. infection. In avocado defense responses initiated after *Fusarium* sp. recognition. Genes associated with signal transduction activation, which are responsive to the recognition of elicitor molecules, such as *LYK3* and *RLK1*, were evidenced. The recognition is also mediated by *R* genes, exemplified by *RPP13*. This recognition and signaling cascades allow the accumulation of reactive oxygen species (ROS) and the activation of various transcription factors (TFs). Activation of these TFs facilitates the involvement of four main processes: microRNA expression, phenylpropanoids biosynthesis, biosynthesis and involvement of phytohormones, and expression of different genes. The main phytohormones involved in pathogenesis responses are AUX, ET, JA, SA, and ABA, the latter being the most represented in hormone-mediated signaling process. ABA can negatively regulate ET and AUX activity by suppressing genes such as *YUCCA4* and *EIN3* and intervene in JA signaling by regulating the *MYC2* gene. ET and JA act synergistically with the involvement of *WRKY33* gene. AUX, on the other hand, is involved, like phenylpropanoids biosynthesis, in root development, where AUX transporter activity is represented by ABC, PIN, and AUX. One process represented is SAR response, which is mediated by crosstalk between phytohormones such as ET, JA, and SA, in addition to the involvement of different genes considered important for optimal SAR responses, such as *ELP2*, *FLD*, *FVE*, and *FMO1*, as well as transporters like EDS5, highlighting the importance of this process as a primary response during the pathogenesis event. The regulatory involvement of microRNAs is reflected at different levels of regulation process. They can intervene in pathogen recognition regulation, as in the case of the miRNA/gene pair *miR157*/*LYK3* and *Ctg0854_RaGOO_5920*/*RLK1*. They can also be involved in phenylpropanoids biosynthesis, as in the case of *miR166*/*MYB4*, and regulate AUX activity with the action of *chr11_RaGOO_17754* on the *YUC5* gene. Both *miR166b* and *chr11_RaGOO_17754* may regulate root development. Finally, SAR may be regulated by the activity of miR166 on the *JAR1* gene and *chr4_RaGOO_33952* on the *PEN3* gene.

In Figure 8, we present a complex network that, in avocado var. *drymifolia*, operates by protein-coding genes, phytohormones, and miRNAs and regulating distinct biological processes, all of them involved in the response to *Fusarium* infection.

## 4. Materials and Methods

### 4.1. Strain of Fusarium sp. and Growth Conditions

The strain INECOL_BM-06 of *Fusarium* sp. was isolated for the first time from the *Xylosandrus morigerus* Reitter ambrosia beetle [166]. Phylogenetically, this strain belongs to the *F*. *solani* Species Complex (FSSC), and both its virulence/pathogenicity, as well as its ability to infect arboreal species of agricultural and forestry interest (cultivars Marsellesa and Oro Azteca from *Coffea arabica* L., citrus as *Citrus* × *latifolia* (Yu.Tanaka) Tanaka and *C*. *sinensis* (L.) Osbeck, *Salix lasiolepis* Benth., and *Populus nigra* L.), have been demonstrated [166]. This strain is part of a collection owned by the Molecular Biology laboratory at INECOL and was provided by Diana Sánchez-Rangel.

The conidia were propagated from 25% glycerol at −80 °C storage into Potato Dextrose Agar (PDA) plates and grown at 28 °C in darkness. After fungal development, a 5 mm × 5 mm square was taken from the PDA plate and placed into a Potato Dextrose Broth (PDB) media. The fungal culture was incubated with continued shaking (180 rpm) at 28 °C for 4 days in semi-dark conditions; after this, the media was forced to 400 mL. The final culture was centrifuged at 12,000 rpm for 30 min. The supernatant was decanted, and the conidia were washed with sterile water. Finally, the conidial suspension (1 × 10^6^ conidia/mL) was used.

### 4.2. Avocado Seedlings

The avocado seeds (var. *drymifolia*) were acquired in a certified nursery (Vivero Salas, Uruapan, Michoacán, México). They were washed with sterile water under aseptic conditions. Then, the avocado seed germination process was accelerated by carefully removing one cotyledon using a scalpel and forceps to avoid damage to the zygotic embryo. The remaining cotyledon-containing embryo, with 3–4 toothpicks inserted, was held on top of the 8 oz containers used to germinate them. Sterile water was added to the empty containers until it covered the middle part of the embryo. Embryos were hydroponically grown in a greenhouse at 28 ± 2 °C with a relative humidity of 80%. Seedlings were monitored weekly for two months, and water was replenished as needed.

### 4.3. Avocado var. drymifolia–Fusarium sp. Hydroponic Pathosystem

We developed a hydroponic infection system with constant air reflux. For this purpose, we used 1000 mL transparent plastic containers (covered with aluminum foil as light-insulating material), a double-outlet air pump with a capacity of 200 L, 0.6-inch air diffusers (air stones), and silicone plastic hoses. Avocado seedlings, previously germinated, were placed inside these containers, which contained enough water to keep roots submerged (500 mL) and maintain the aerial part of the plant (the shoot) outside the containers (Figure S3). This hydroponic system allowed increased root contact with the inoculated spores, avoiding its sedimentation. Four sampling points over time were chosen to analyze the infection/disease progress: 1-, 7-, 14-, and 21-dpi (Figure 1). As appropriate, four plants, either infected or uninfected (control) treatments, were used to represent each sampling point. Infected plants were inoculated with *Fusarium* sp., where the suspension (500 mL) on the hydroponic system was maintained constantly at a concentration of 1x10^6^ conidia/mL. Conidia suspension and sterile water were replaced every three days until the end of the experiment (21 dpi). The avocado var. *drymifolia* plants were grown in a culture chamber (Thermo Fisher Scientific, model 3768) with a 22/24 °C temperature regime, a photoperiod of 16 h/8 h (light/dark), relative humidity of around 60%, and a light intensity of 700 µmol m^−2^s^−1^. For each sampling time, a separate collection of the tissues (leaf, stem, root) was made, instantaneously frozen with liquid nitrogen, and stored at −80 °C until use.

### 4.4. Avocado var. drymifolia Genome Assembly, Base Correction, and Gap Filling

In contrast with the avocado genome from the Hass cultivar, until this work was conducted, the genome available in the public databases for the *drymifolia* variety was highly fragmented and filled with sequence gaps [34]. To improve it, first, the available contigs from the *drymifolia* variety were ordered, oriented, and merged based on the reference genome (the Hass cultivar genome). RaGOO software v1.1 [167] was used for this purpose. Then, to correct erroneous bases, fill gaps, and correct false segmental duplications, we used the software Pilon v1.2 [168] and an Illumina high-quality reads dataset (134,241,527 single-end reads and 161,105,050 paired-end reads) representing a deep coverage (50x); this was repetitively aligned (five iterations) against the “new” draft from avocado var. *drymifolia* genome.

### 4.5. Gene Models Prediction and Annotation

Prior to gene annotation, repetitious sequences in the avocado genome were masked using RepeatMasker v4.1.1 [169] with the soft masking option. The TE from the avocado genome (Hass cultivar) reported by Rendon-Anaya et al. [34] and *de novo* predicted with the REPET v2.2 package [170] were used as reference sequences. In addition, as transcriptional evidence, we include the RNA sequencing (RNA-seq) reads generated for the study presented here, and datasets from several previously published avocado transcriptomic studies [45,171,172,173,174] were downloaded from the NCBI Sequence Read Archive (SRA) database (Bioprojects: PRJNA253536, PRJNA282441, PRJNA551035, PRJNA551035). Finally, as a dataset used for training and refinement of the gene models resulting from the annotation process, around 500,000 avocado protein sequences, all of them manually curated and translated from unigenes (or unique transcripts) resulting from the assembled transcriptomes mentioned above, were combined in a single homemade database. Quality and adapter-trimmed RNA-seq reads were aligned to the masked genome using HISAT2 v2.1.0 [175]. Annotation of protein-coding genes in the avocado var. *drymifolia* genome was conducted using a combination of homology-based prediction, *de novo* prediction, and transcriptome-based prediction methods using BRAKER2 v2.1.6 [176], which combines the predictions made by AUGUSTUS v3.4.0 [177,178,179,180] and GeneMark-ES/ET v4.65 [181,182] to ensure the integration of high-quality gene models into the result. Benchmarking sets of Universal Single-Copy Orthologs (BUSCO [183] were used to assess the completeness of genome annotation. The homolog genes were identified using the Best BLAST Hit (BBH) method and the Basic Local Alignment Search Tool (BLAST v2.9.0; [184]). A database containing the proteins predicted in twenty angiosperm plant species whose genomes had been sequenced completely was used as a reference. Like previous studies involving avocados as a study model, these plant species selected as reference obey a distribution of representative clades along the angiosperm plant phylogeny [34,45].

### 4.6. RNA-Seq Library Preparation and Sequencing

As the used hydroponic system favors all-time plant–pathogen interaction in the root system, allowing even *Fusarium* sp. INECOL_BM-06 to colonize the root tissue during active plant growth, we decided to generate, at least for the study presented here, RNA-seq libraries only from the roots harvested from infected and uninfected (control) plants at 1-, 7-, 14-, and 21 dpi. Once harvested, root tissue was frozen with liquid nitrogen and ground with a pestle and mortar into a fine powder. Then, RNA was isolated from 100 ng of pulverized tissue using the Plant/Fungi Total RNA Purification Kit (Norgen Biotek Corp, Thorold, ON, Canada) according to the manufactured instructions. Concentration and purity (260:280/260:230 absorbance ratios) of the isolated total RNAs were measured on a NanoDrop 2000c spectrophotometer (Thermo Scientific, Waltham, MA, USA), while RNA integrity was evaluated using capillary electrophoresis by a Bioanalyzer 2100 System (Agilent Technologies, Santa Clara, CA, USA) and agarose electrophoresis. RNA-seq libraries were prepared and sequenced at the Laboratorio de Servicios Genómicos (LABSERGEN) from the Advanced Genomics Unit (UGA, before LANGEBIO) and were generated as follows:

For mRNA sequencing: high-quality RNA (≈500 ng) was processed with the TruSeq RNA Sample Prep Kit version 2.0, following the manufacturer’s instructions. Briefly, using poly-T oligo-attached magnetic beads, mRNA was poly-A-selected to deplete the ribosomal RNA fraction. The cleaved mRNA fragments were reverse transcribed into cDNA, and once the ends were repaired, the Illumina dual-index adapters were ligated onto them. Each library (sixteen in total, *i.e.*, two independent biological replicates from infected and uninfected plants at 1-, 7-, 14-, and 21 dpi) was independently labeled with a specific multiple. Then, 250–350 bp fragments were excised with Agencourt^®^ AMPure XP beads (Beckman Coulter, Brea, CA, USA) and amplified by PCR. After purification, the libraries’ quality was checked using a Bioanalyzer 2100 (Agilent Technologies, Santa Clara, CA, USA). mRNA sequencing was then performed on a NextSeq500 (Illumina, San Diego, CA, USA) platform to generate 150 bp paired-end reads (300 cycles).

For miRNA sequencing: small-RNA libraries were prepared using the TruSeq Small RNA Sample Prep Kit (Illumina, San Diego, CA, USA). From the high-quality total RNAs (≈5 μg), small RNA fragments ranging from 18–30 nt were isolated, purified, and subsequently ligated to 3′ and 5′ ends adaptors. Sequentially, RNAs were reverse transcribed into cDNA and then PCR amplified (fifteen cycles). In contrast with mRNA samples, we prepared only two libraries for the miRNA sequencing, one from infected and the other for uninfected plants. An equimolar quantity of the small RNA fragments purified/isolated from each sampling point (1-, 7-, 14-, and 21 dpi) was mixed to prepare each of them. Both libraries (infected and uninfected) were tested by gel electrophoresis, and bands corresponding to miRNA insertion were cut and eluted. After ethanol precipitation and washing, the miRNA libraries were quantified and sequenced using the NextSeq500 (Illumina, San Diego, CA, USA) platform (single-end reads, 50 cycles).

### 4.7. Differentially Expressed Avocado Genes Responsive to Fusarium sp. Infection and GO Enrichment Analysis

To identify the protein-coding genes that, in avocado var. *drymifolia*, respond to *Fusarium* sp. infection, first, an expression profiles matrix was created containing each of the genes predicted in the genome (rows) and the expected counts (EC) values calculated for both conditions assayed (infected an uninfected) on each of the selected sampling point (columns). The EC values (and also reads-per-million (RPM) values) were obtained as results of the analysis performed with the RSEM (RNA-seq by Expectation Maximization) software v1.3.1 [185]; they represent relative expression levels and are calculated by a maximum likelihood estimation approach as well as posterior mean estimates with 95% credibility intervals, once the number of high-quality reads that map to each gene has been counted. We chose Bowtie2 [186] as the mapper used by RSEM v1.3.1. To increase the number of reads mapped to each gene in the reference genome, we use extended gene models, *i.e.*, we add 250 bp downstream and upstream to coding regions. The length of 5′ and 3′ untranslated regions (UTRs) correspond to the average length estimated from close to 25% of the genes annotated in the genome and in which the UTR regions were supported by transcriptional evidence. Notice that this average length is consistent with a previous report in which it has been estimated that 5′ UTRs are roughly constant over diverse taxonomic classes and range between 100 and 200 nucleotides. In contrast, the average length of 3′ UTRs is much more variable, ranging from about 200 nucleotides in plants and fungi [187].

Pairwise comparisons (infected versus uninfected (control) treatments at each sampling point (1-, 7-, 14-, and 21 dpi)) were performed in order to identify differentially expressed genes (DEGs). EdgeR Bioconductor package [188] was used for this purpose; thus, using the TMM (Trimmed Mean of M-values) method [189], the data were normalized based on a theoretical negative binomial distribution. The selection of DEGs in response to infection was performed considering a fold change values greater than 2 or less than 0.5 (Log_2_FC = ± 1) and False Discovery Rate (FDR)-corrected *p*-values (adjusted *p*-value) ≤ 0.05 as threshold. In addition, *k*-means clustering analysis was performed; that is, by an unsupervised method, the DEGs were clustered based on their expression pattern observed over time. Spearman’s correlation coefficient test was conducted to evaluate statistical dependence between the distinct sampling points. The appropriate number of clusters (k) in this *k*-means analysis was calculated using the sum of squared error and the elbow method [190]. The expression profile of the genes belonging to each cluster was subsequently plotted using ggplot2 and reshape libraries for R. Finally, using the ShinyGo v.0.76.3 [191] web server (http://bioinformatics.sdstate.edu/go/, accessed on 11 July 2023), an enrichment of the functional categories was generated from those DEGs in order to provide information on the biological processes in which they participate.

### 4.8. Identification of miRNAs and Their Targets That Respond to Fusarium sp. Infection in Avocado var. drymifolia

Single-end reads obtained from miRNA libraries were filtered and trimmed to remove low-quality reads and sequencing adaptors. A Python-based script (qualityControl.py, from https://github.com/Czh3/NGSTools accessed on 3 October 2022) and the Cutadapt software v2.5 [192] were used for this purpose, establishing a threshold sequence window of 20–30 nt per read. Unique sequences represented by ≥10 RPM were considered to be significantly expressed above the background noise [193] and thus selected for further analyses. MiRNA identification was carried out using miRdeep2 software v0.1.3 [52] with the following settings: (*i*) reads map perfectly to the reference avocado var. *drymifolia* genome, (*ii*) cut off -v 1, and (*iii*) employing the “-s option” using all mature sequences from miRBase database (https://www.mirbase.org/; accessed on 3 October 2022) [194,195,196]. Folding analyses of the novel pre-miRNAs detected in nodule fluids were carried out using the RNAfold Vienna package with default settings [197]. To identify miRNAs responsive to *Fusarium* sp. infection, avocado pre-miRNAs were used as a reference, and the high-quality reads from both infected and uninfected plants, processed according to the pipeline mentioned before, were independently mapped onto them. Once more, Bowtie2 v2.3.5.1 and RSEM v1.3.1 were the software packages used to conduct this process. The EC values from both treatments (infected vs. uninfected) were compared to each other, and those pre-miRNAs differentially expressed (Log_2_FC values greater than 1) in a significative way were identified by Fisher’s exact test and the Bonferroni correction for multiple testing (*p*-value ≤ 0.05, α = 0.1; probability of Error Type I). Finally, miRNA targets were computationally predicted using the psRNATarget tool [198], with the default parameters and a maximum of two allowed mismatches. Mature miRNA produced from precursor microRNAs (pre-miRNAs) that were differentially expressed (upregulated) were the “query,” while extended gene models of downregulated differentially expressed genes (see results) were used as target candidate genes.

## 5. Conclusions

Despite the knowledge about defense response in plants against fungal pathogens, many molecular players involved in the immune response in new pathosystems remain uncharacterized. In this work, we contributed evidence about the genes and miRNAs that differentially expressed in avocado, an ancient arboreal plant during the infection of *Fusarium* sp. Our research revealed that the principal responses modulated by miRNA intervention to avoid infection include carbohydrate and polysaccharide metabolism and auxin biosynthesis. The enrichment of protein-genes related to root development, defense responses, and phytohormone signaling underscores the complexity of the plant’s reaction to pathogen invasion and, finally, through comprehensive genome assembly, transcriptomic analysis, and miRNA prediction, we have established a robust framework to contribute significantly to the understanding of avocado responses to fungus pathogen infection and provides valuable insights for future research on plant–pathogen interactions in an important commercial crop.

## Figures and Tables

**Table 1:** DEmiRNAs responsive to *Fusarium* sp. in avocado var. *drymifolia* root. The table shows the associated *p*-values, the fold change calculated under Fisher’s exact test, and the metrics of its predicted target genes. From left to right, the table includes the total number of avocado var. *drymifolia* genes predicted as target genes, those target genes annotated that find and homolog in *A*. *thaliana*, and finally those target genes that were annotated and unique for each DEmiRNA.

Identifier (Name)	*p*-Value ^1^	Log_2_(FC)	# of Predicted Target Genes		
			Predicted	Annotated	Unique
*Ctg0811_RaGOO_5704*	2.77 × 10^−6^	3.5866	58	35	33
*Ctg0854_RaGOO_5920*	4.75 × 10^−2^	1.2426	122	82	76
*miR157d-12*	4.14 × 10^−6^	18.4134	94	59	53
*miR166b-19*	8.40 × 10^−10^	0.8181	74	46	32
*miR166g-17*	2.24 × 10^−14^	3.1221	68	50	39
*chr11_RaGOO_17754*	4.91 × 10^−2^	1.0403	79	57	51
*chr3_RaGOO_29551*	3.35 × 10^−2^	1.0000	56	40	37
*chr4_RaGOO_33952*	2.73 × 10^−2^	0.9487	72	42	39

^1^ The *p*-value reflects the degree of data compatibility with the null hypothesis. A *p*-value ≤ 0.05 was considered significant.

## Data Availability

The datasets supporting the findings of this study can be publicly obtained at NCBI GenBank. The highly fragmented old version from *Persea americana* var. *drymifolia* genome assembly, which was used as input in the improvement process, can be downloaded by accession GCA_008033785.1. Short reads used to improve the genome assembly by its iterative mapping belong to Bioproject ID: PRJNA508502. Biosamples: SAMN10523736. The new version from the *Persea americana* var. *drymifolia* genome assembly and gene models are available on CoGe (http://genomevolution.org/CoGe/; accession ID: 68395, uploaded 6 June 2024). RNA-seq raw sequence reads of infected and uninfected avocado seedlings were submitted to the NCBI Sequence Read Archive (SRA) database and belong to Bioproject ID: PRJNA1149708.

## Supplementary Materials

The following supporting information can be downloaded at: https://www.mdpi.com/article/10.3390/plants13202886/s1, Figures S1: Impact of *Fusarium* sp. infection in development and growth of some organs of avocado var. *drymifolia* seedlings; Figure S2: Stem-loop hairpin secondary structures of potential new miRNA precursors (pre-miRNA) identified in avocado var. *drymifolia* genome; Figure S3: Hydroponic-based system for growing avocado var. *drymifolia* seedlings in which *Fusarium* sp. infection symptoms can be evaluated over time. Table S1: Predicted genes in avocado var. *drymifolia* genome and its annotation performed by homology with *A. thaliana* genome; Table S2: Assembly metrics of available and improved version of avocado var. *drymifolia* genome; Table S3: Statistics of sequence masking and TE identification in the avocado var. *drymifolia* genome; Table S4: A summary of the Illumina sequencing dataset generated in this study from roots of infected and uninfected avocado seedlings (avocado var. *drymifolia*); Table S5: Clusters of avocado var. *drymifolia* differentially expressed genes (DEGs) with similar expression patterns; Table S6: GO-terms assigned to avocado var. *drymifolia* DEGs; Table S7: Hormone-mediated processes in C4 and C6; Table S8: Predicted novel avocado var. *drymifolia* miRNAs, together with their pre-miRNA sequences; Table S9: Prediction of target genes of avocado var. *drymifolia* miRNAs responsive to *Fusarium* sp. infection; Table S10: Gene ontology (GO) functional enrichment analysis of identified targets of DEmiRNAs; Table S11: Target genes involved in SAR responses and phytohormone metabolism.
