# Supplementary material for: Genome-Wide Transcriptional Response of Avocado to Fusarium sp. Infection
Source: Plants (Basel). 2024 Oct 15;13(20):2886. doi: 10.3390/plants13202886 (PMC11511450; doi:10.3390/plants13202886)
Supplement: Supplementary file 1 [file plants-13-02886-s001.zip › plants-3192890-Supplementary Figures (S1-S3).pdf]

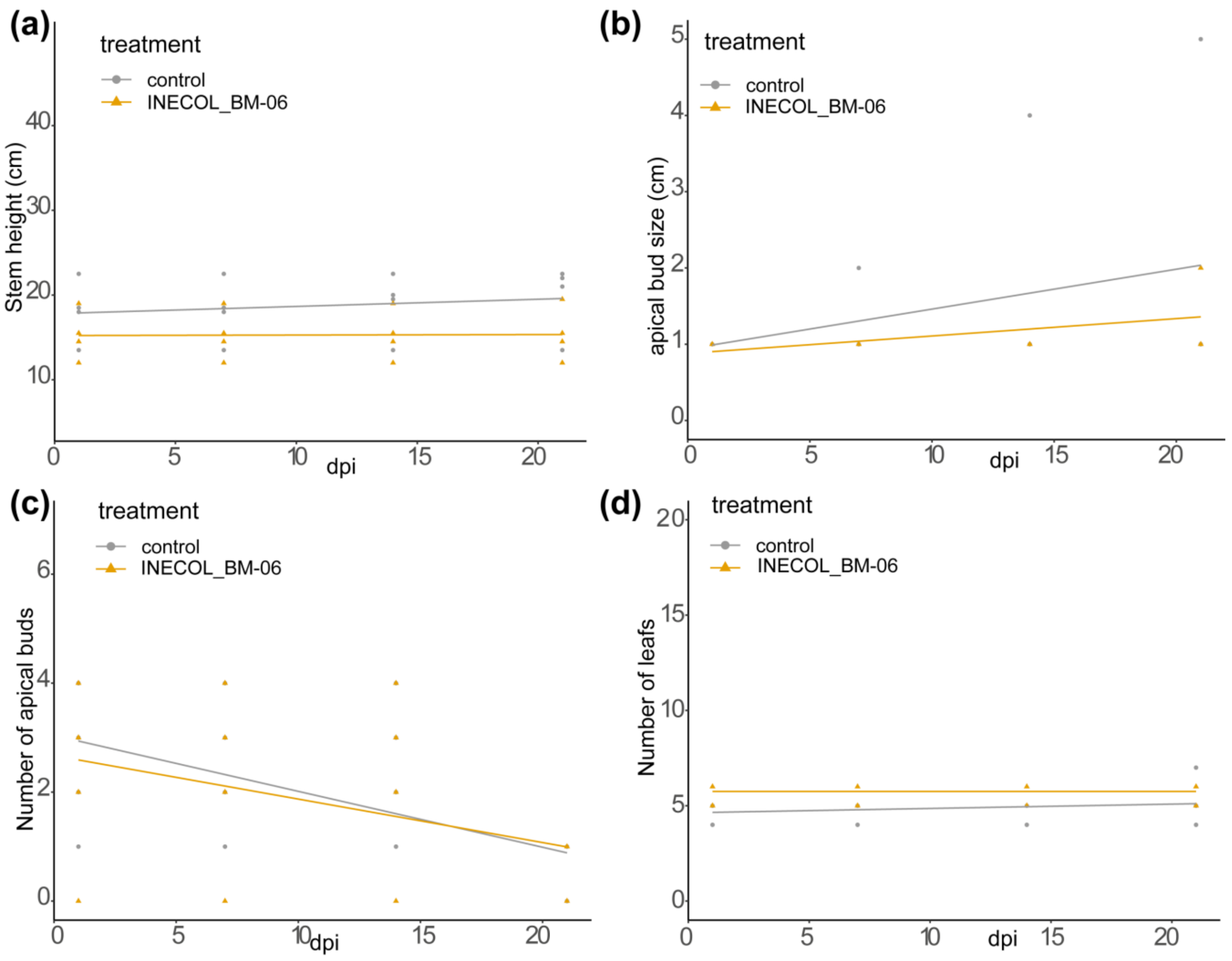

Figure S1. Impact of *Fusarium* sp. infection in development and growth of some organs of avocado var. *drymifolia* seedlings. The organs and traits analyzed comprise seedling height or stem length (**a**), the size and number of apical buds (**b** and **c**, respectively), and the number of leaves in seedlings (**d**). Lines in grey color indicate uninfected plants; yellow color indicates infected plants.

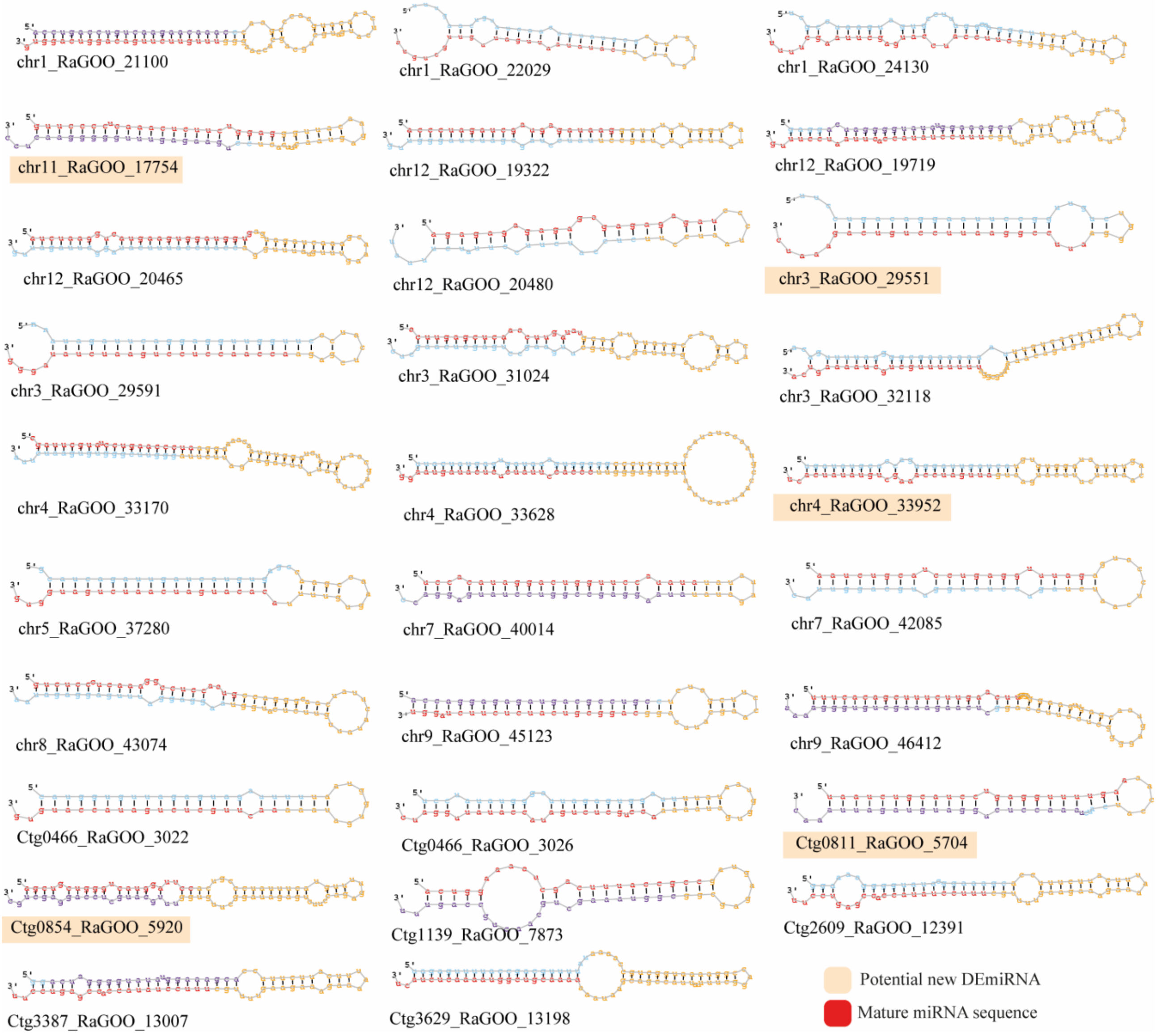

Figure S2. Stem-loop hairpin secondary structures of potential new miRNA precursors (pre-miRNA) identified in avocado var. *drymifolia* genome. Those Identifiers assigned and highlighted in yellow correspond to those differentially expressed miRNAs in response to *Fusarium sp.* infection (DEmiRNAs). In the hairpin structure, mature miRNA is shown in red.

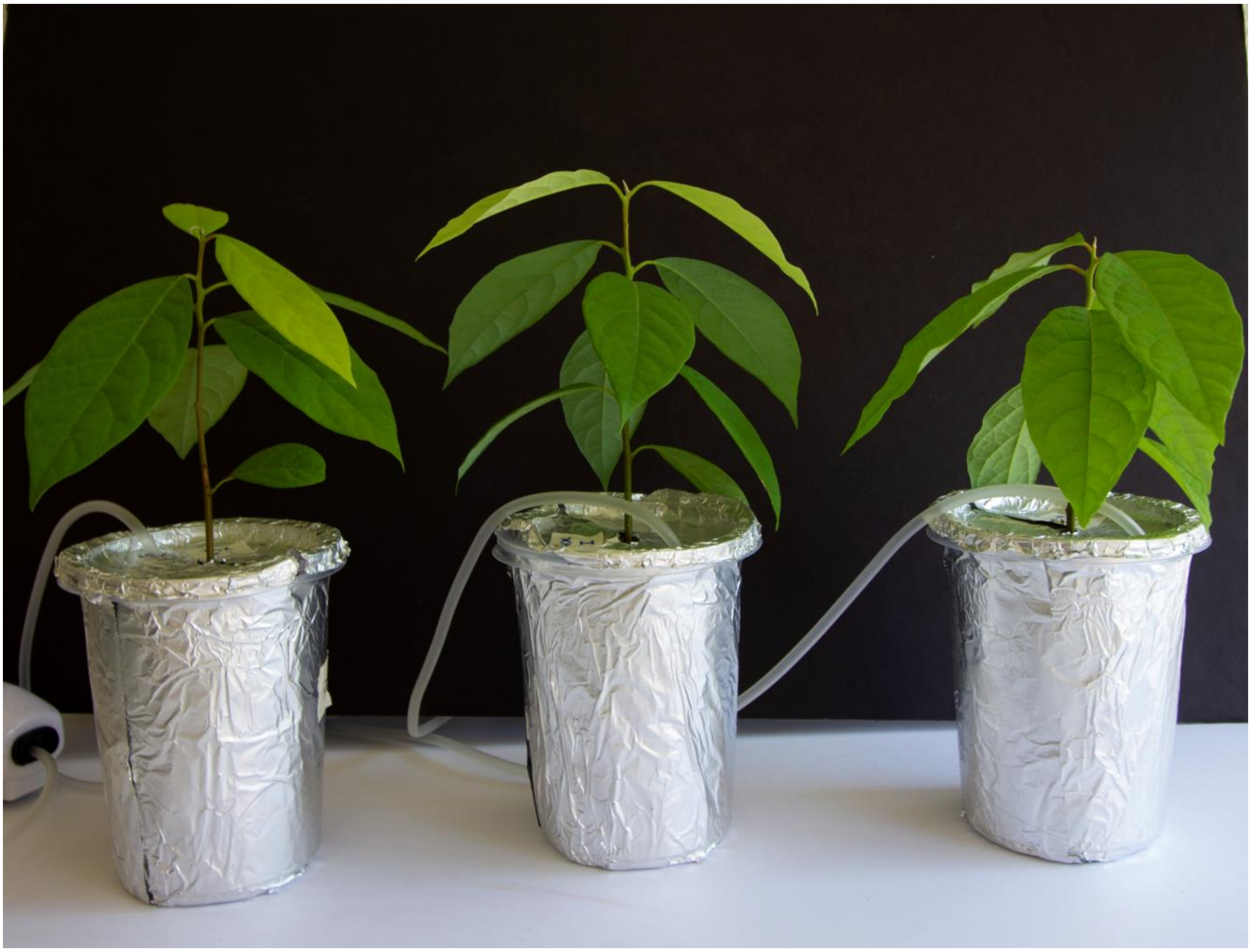

Figure S3. Hydroponic-based system for growing avocado var. *drymifolia* seedlings in which *Fusarium* sp. infection symptoms can be evaluated over time. The infection is achieved after water is replaced by conidial suspension ($1x10^6$ conidia/mL), which is refreshed every three days until the end of the experiment (21 dpi in this study).
